# A novel variation in *RSPO4* causing nonsyndromic congenital nail disorder-4 in a Chinese patient

**DOI:** 10.3389/fped.2025.1592954

**Published:** 2025-06-20

**Authors:** Qiang Zhang, Qi Yang, Xunzhao Zhou, Sujie Zhang, Jingsi Luo

**Affiliations:** ^1^ Guangxi Key Laboratory of Birth Defects Research and Prevention, Guangxi Key Laboratory of Reproductive Health and Birth Defects Prevention, Maternal and Child Health Hospital of Guangxi Zhuang Autonomous Region, Nanning, China; ^2^ Department of Genetic and Metabolic Central Laboratory, Maternal and Child Health Hospital of Guangxi Zhuang Autonomous Region, Nanning, China; ^3^ Department of Child Health Care, Maternal and Child Health Hospital of Guangxi Zhuang Autonomous Region, Nanning, China; ^4^ Guangxi Clinical Research Center for Pediatric Diseases, Maternal and Child Health Hospital of Guangxi Zhuang Autonomous Region, Nanning, China; ^5^ Hematology Laboratory, Sheng Jing Hospital of China Medical University, Shenyang, China

**Keywords:** congenital nail disorder, *RSPO4* gene, novel variation, whole-exome sequencing, genetic analysis

## Abstract

**Background:**

Non-syndromic congenital nail disorder type 4 (OMIM: 206800) is a rare autosomal recessive condition characterized by severe hypoplasia or complete absence of fingernails and toenails. This disorder results from variants in the *RSPO4* gene (OMIM: 610573) located on chromosome 20p13.

**Objective:**

This study aimed to assess the potential pathogenicity of a novel *RSPO4* variant identified in a Chinese patient and to explore the phenotypic and molecular genetic characteristics of Non-syndromic congenital nail disorder type 4.

**Methods:**

Whole-exome sequencing was performed in the proband to identify candidate variants. Sanger sequencing validated the variant and determined its origin. In silico prediction tools were used to evaluate the variant's functional impact. Pathogenicity classification followed the American College of Medical Genetics and Genomics guidelines. A systematic literature review was conducted to collate previously reported cases.

**Results:**

A novel frameshift variant in the *RSPO4* gene—c.268dupA/p.Cys91fs*6—was identified and classified as likely pathogenic based on the American College of Medical Genetics and Genomics guidelines. The clinical presentation of the patient was consistent with the diagnostic criteria for Non-syndromic congenital nail disorder type 4. Literature review confirmed that Non-syndromic congenital nail disorder type 4 primarily manifests as generalized nail dysplasia without associated hair, dental, or skeletal abnormalities.

**Conclusion:**

This study expands the known genetic spectrum of Non-syndromic congenital nail disorder type 4 and enhances the understanding of phenotypic characteristics associated with *RSPO4* variants. The findings have potential implications for improving variant-based screening, genetic diagnostics, and molecular insights into *RSPO4*-related disorders.

## Introduction

Isolated nonsyndromic congenital anonychia/hyponychia is a rare condition, typically inherited in an autosomal recessive manner with variable expression within families (1). Nail phenotypes range from complete absence to reduced nail field size with absent or rudimentary nails (2). Blaydon et al. (3) and Bergmann et al. (4) identified variants in the *RSPO4* gene in families with autosomal recessive isolated congenital anonychia, also known as Non-syndromic Congenital Nail Disorder Type 4 (NDNC4). Other nonsyndromic congenital nail disorders include NDNC2 (5), NDNC3 (6, 7), NDNC5 (8), NDNC6 (9), NDNC7, NDNC8 (10, 11) and NDNC9 (12) (Supplementary Table S1). R-spondin-4, encoded by the *RSPO4* gene, is a secreted protein that regulates β-catenin signaling (13). The 224-amino acid human R-spondin-4 shares structural similarities with other R-spondin family members, including an N-terminal signal peptide, two furin-like domains, a thrombospondin type-1 domain, and a C-terminal low-complexity region rich in positively charged amino acids (14). *RSPO4* expression is notably weak in adult tissues and tumors (15). R-spondin-4 is active in bone tissue and involved in limb formation, particularly in nail development at the tips of fingers and toes (16–18). It enhances Wnt signaling activity by acting as a ligand for LGR4-6 receptors, triggering the canonical Wnt signaling pathway to increase target gene expression and regulating both canonical and non-canonical Wnt signaling by inhibiting ZNRF3 (19–21). The Wnt/β-catenin signaling pathway regulates fundamental cellular processes such as proliferation, adhesion, and differentiation (22–24) and is crucial for nail growth and development during early stages (25). *RSPO4* gene variants, primarily located in the furin-like domains, result in nonfunctional or hypofunctional proteins (26–28), preventing R-spondin-4 from participating in the Wnt signaling pathway and leading to abnormal or absent nail development. To date, only 17 RSPO4 variants (affecting 22 families) have been reported worldwide (Table 1), all causing congenital anonychia. In this study, we report a case of a Chinese female patient with reduced nail size and swollen nail matrix linked to a novel *RSPO4* variant on the 20p13 region of the human chromosome.

**Table 1:** Summary of clinical and molecular features of all patients with RSPO4 variants.

Clinical features	F1 （^2^）	F2（^2^）	F3（^3^）	F4–5（^3^, ^18^）	F6–7（^3^）	F8–9（^3^）	F10（^3^）	F11（^4^）	F12（^14^）	F13–16 （^3^, ^17^）	F17 (17)	F18 (17)	F19 (29)	F20 (20)	F21 (18)	F22 (30)	Our study	Statistics
Country	Kazakh	Turkish	Finnish	Pakistani	Irish/English	Pakistani/Irish	Pakistani	German	Pakistani	Pakistani/English	Pakistani	Pakistani	Turkey	Pakistani	Pakistani	Lebanese	China	9 country
Consanguineous marriage	–	+	+	+	–	+	+	–	+	+	+	+	+	+	+	+	–	18/23 consanguineous marriage
Phenotype	Absence of finger- and toenails. The skin in the region of the absent nails was normal, and the nail bed, nail matrix, and fold were present in all fingers and toes	Absence of finger- and toenails	The nail field is reduced in size, the nail plate is absent, the nail matrix is swollen. the nail bed has protective hyperkeratosis	The nail field is reduced in size, the nail plate is absent, the nail matrix is swollen	The nail field is reduced in size, the nail plate is absent, the nail matrix is swollen	The nail field is reduced in size, the nail plate is absent, the nail matrix is swollen. the nail bed has protective hyperkeratosis	The nail field is reduced in size, the nail plate is absent, the nail matrix is swollen. the nail bed has protective hyperkeratosis	The nail field is reduced in size, the nail plate is absent, the nail matrix is swollen	Complete absence of fingernails and toenails. The skin in the region of the absent nails was normal and the nail bed and nail matrix were present in all fingers and toes	Complete absence of the nail plate and matrix, with only the nail bed present, or hyponychia, with some remnants of rudimentary, fragile nail plates	Complete absence of the nail plate and matrix, with only the nail bed present, or hyponychia, with some remnants of rudimentary, fragile nail plates	Complete absence of the nail plate and matrix, with only the nail bed present, or hyponychia, with some remnants of rudimentary, fragile nail plates	Nail deformities and anonychia in her toenails	Absence of fingernails and toenails	Absence of fingernails and toenail	Total anonychia involving the fingernails	Growth retardation short stature, nail dysplasia, hyperhidrosis	23/23 nail dysplasia, hyperhidrosis
Homozygous or compound heterozygous	Homozygote	Homozygote	Homozygote	Homozygote	Compound heterozygous	Homozygote(Pakistan)/compound heterozygous (Irish)	Homozygote	Compound heterozygous	Homozygote	Homozygote(Pakistani)/compound heterozygous (English)	Homozygote	Homozygote	Heterozygous (proband and siblings) and Homozygote (mother and her siblings)	Homozygote	Homozygote	Homozygote	Homozygote	20/23 homozygote
Exon	3	2	2	3	3	1 and 3	2	2	2	1 and 3	1	2	1	1	2	2	2	exon 1 to exon 3
Mutation (NM_001029871.4)	c.301C>T (p.Gln101Ter)	c.190C>T (p.Arg64Cys)	c.194A>G (p.Gln65Arg)	c.353G>A (p.Cys118Tyr)	c.319T>C (p.Cys107Arg); c.284G>T (p.Cys95Phe)	c.79+1G>A（also known as IVS1+1G>A） (Pakistani/Irish); c.319T>C (p.Cys107Arg)(Irish)	c.95-110del (p.Gly32AlafsTer189)	c.218G>A (p.Cys73Tyr); c.98dup (p.Asn34GlnfsTer63) [also known as c.92_93insG (p.Leu31GlufsTer45)]	c.199G>C (p.Gly67Arg)	c.80-1G>A (also known as IVS1-1G>A) (Pakistani/English); c.284G>T (p.Cys95Phe) (English)	c.-9_17del	c.269-1G>A（also known as IVS2-1G>A）	c.79+1G>A	c.18C>A (p.Cys6Ter)	c.178C>T (p.Arg60Trp)	c.164-165TC>AA (p.Phe55Ter)	c.268dupA (p.Cys91MetfsTer6)	18 variants
Types of variation	Nonsense	Missense	Missense	Missense	Missense	Splice site/missense	Frameshift	Missense/frameshift	Missense	Splice site/missense	Deletion	Splice site	Splice site	Nonsense	Missense	Nonsense	Frameshift	Lof:missense = 10:8

## Materials and methods

### Next-generation sequencing

Genomic DNA was extracted from the patient's peripheral blood. Target enrichment was performed using the Agilent SureSelect Clinical Research Exome V2 Kit (Agilent Technologies, Santa Clara, CA, USA) to construct the sequencing library. Sequencing was performed on the Illumina HiSeq2500 System (Illumina, San Diego, CA, USA). The resulting sequencing reads were aligned to the human reference genome (GRCh37/hg19) with Burrows-Wheeler Aligner (BWA) software (v 0.7.17). Variant calling and annotation were performed using the Genome Analysis Toolkit (GATK), with additional annotation refinement and variant prioritization conducted using TGex software (v 5.7, LifeMap Sciences).

### Sanger sequencing validation

Candidate variants identified using TGex were validated through Sanger sequencing. Primers (*5*′-*CCCTGCTTCTTTTTCACCTC-3*′ and *5*′-*ACCATCTCTCTCTCCCTTTC-3*′) targeting the *RSPO4* c.268dupA/p.Cys91fs*6 locus were designed using Oligo7 (v 7.60, Molecular Biology Insights) and synthesized by Sangon Biotech. PCR amplification was performed under standard conditions (58°C annealing, 35 cycles), and products were sequenced on an ABI 3730XL analyzer (Thermo Fisher Scientific).

### Bioinformatic analysis and verification of observations

We utilized several predictive tools to evaluate the functional effects of the identified variants, including AutoPVS1 (https://autopvs1.genetics.bgi.com), MutationTaster (https://www.mutationtaster.org), ENTPRISE-X (http://cssb2.biology.gatech.edu/entprise-x), and RDDC (https://rddc.tsinghua-gd.org). Together, these tools provide a comprehensive functional analysis from multiple perspectives. For variant classification, we followed the guidelines established by the American College of Medical Genetics and Genomics guidelines (ACMG/AMP) (31).

## Results

### Clinical presentation

**Figure 1:**
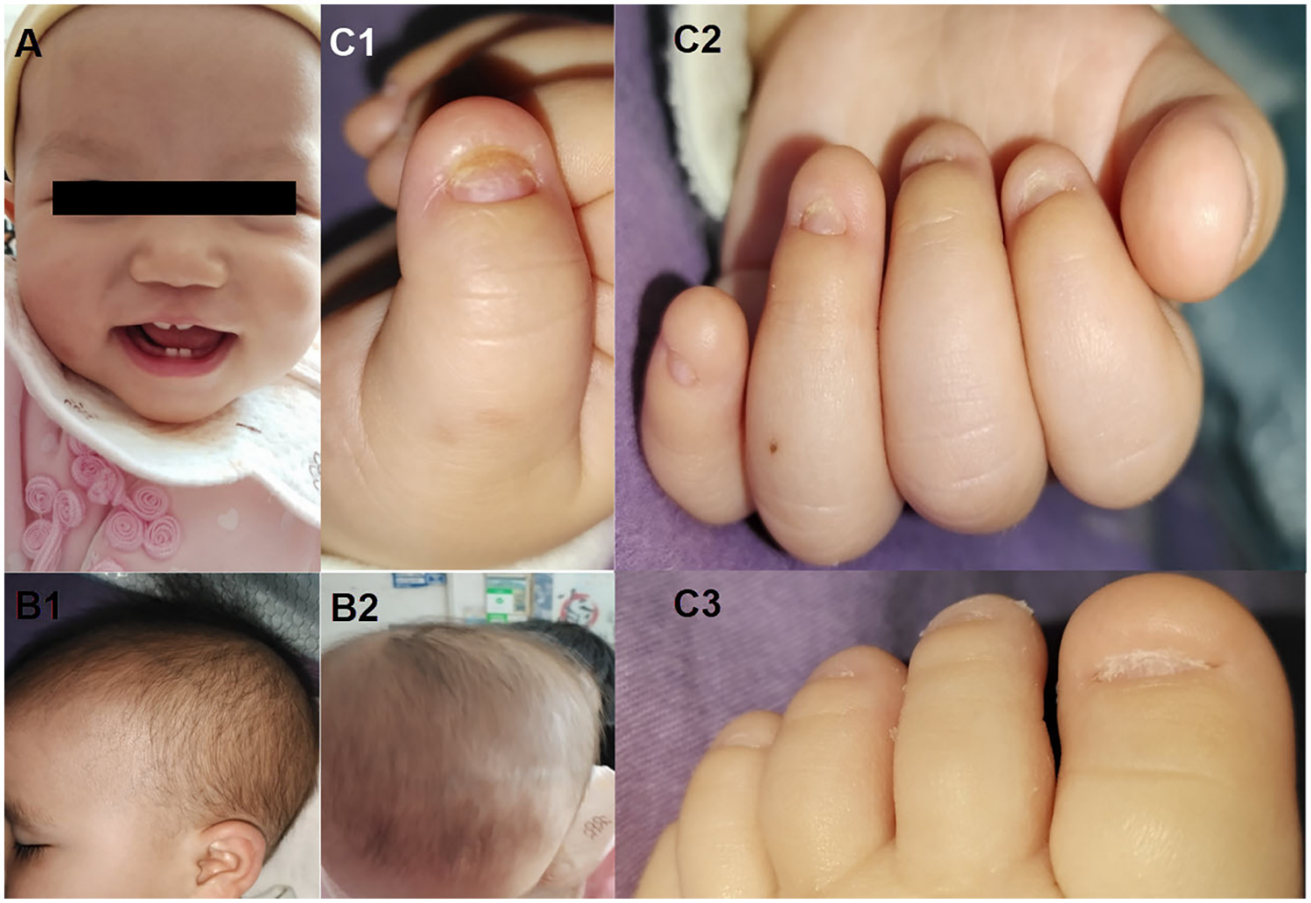
Clinical features of the patient with an *RSPO4* pathogenic variant. **(A)** Normal tooth development with no abnormalities observed. **(B1–B2)** Normal hair distribution (no alopecia). **(C1–C3)** Onychodysplasia (characterized by: reduced nail field size; absence of the nail plate; swelling of the nail matrix with hyperkeratosis).

A 9-month-old Chinese female presented with hypoplastic fingernails and toenails accompanied by postnatal growth retardation [height: 65 cm (-2 SD)]. The proband showed no abnormalities on prenatal ultrasound and was born at full term following a spontaneous vaginal delivery, with no evidence of perinatal asphyxia, hypoxia, or birth trauma. At birth, weight was 2.5 kg (lower end of the normal range), length was 48 cm (−1 SD), and head circumference measured 33 cm (within normal limits). There is no history of consanguinity in the parents, and the family denies any hereditary conditions including ectodermal dysplasia or other genetic disorders. Physical examination of the patient revealed the following: height 65 cm (below the WHO growth standard Z-score of −2 SD, indicating growth retardation), weight 8.5 kg (within the normal range), and head circumference 44.5 cm (within the normal range). Skin turgor was normal (skin fold retraction time <2 s), with no primary or secondary skin lesions such as rashes, blisters, or nodules observed. However, hand-foot hyperhidrosis was noted. Total nail dysplasia affected all fingernails and toenails, characterized by hypoplastic nail beds, absent nail plates, and nail matrix edema (see Figures 1C1–C3). Scalp hair distribution and texture were normal, with no alopecia or scaling. The orofacial region showed no structural abnormalities such as cleft lip or palate, intact oral mucosa, normal tooth morphology (see Figures 1A,B1,B2). Examinations of the cardiovascular system, lungs, and abdomen revealed no abnormalities. The musculoskeletal system was normal. Neurological examination showed symmetric physiological reflexes and negative pathological reflexes. Laboratory tests showed normal complete blood count, biochemistry, and electrolytes, ruling out the possibility of metabolic diseases.

### Genetic analysis of whole-exome sequencing

Genetic factors play a significant role in ectodermal dysplasia. To elucidate the molecular basis, we performed whole-exome sequencing (WES) using the Agilent SureSelect kit, achieving 99.0% target coverage at ≥20× depth. Among 123,548 variants, TGex software (LifeMap Sciences) prioritized five candidate genes (*PLEC, FZD2, EDAR, RANBP2, and RSPO4*) based on phenotype–genotype correlations from the OMIM database. After filtering for population frequency (gnomAD <0.1%), Inheritance pattern/variant origin, and predicted pathogenicity, the homozygous frameshift variant c.268dupA/p.Cys91fs*6 in the *RSPO4* gene was identified as the most likely causative variant.

**Figure 2:**
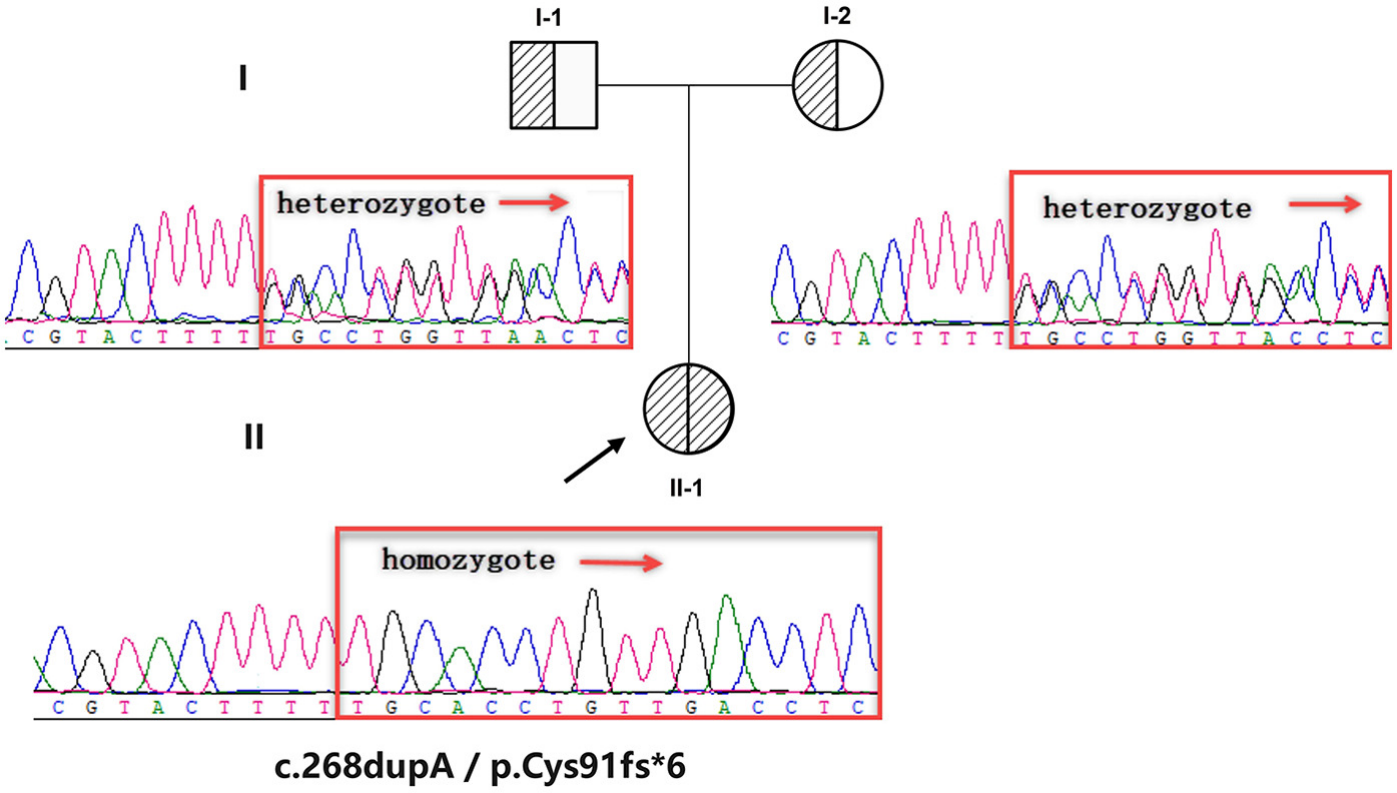
Pedigree and sanger sequencing results. The pedigree depicts a nuclear family structure, with individual II-1 (an affected female) represented by a half-shaded circle. Squares denote males and circles females. Sanger sequencing chromatograms of key family members demonstrate heterozygous genotypes in both parents (I-1 and I-2), whereas the proband (II-1) carries a homozygous variant.

**Figure 3:**
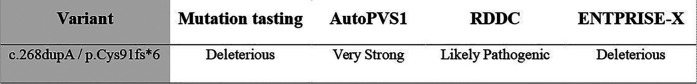
Functional impact of the *RSPO4* c.268dupA/p.Cys91fs*6 variant*.* In silico analyses: Multiple computational tools consistently predicted this variant to be damaging or pathogenic, further supporting its potential deleterious effects on protein function.

Sanger sequencing confirmed the authenticity of the variant, and pedigree analysis revealed that both parents are heterozygous carriers, indicating the homozygous variant was inherited in a biparental manner (Figure 2). The c.268dupA variant is absent in public databases (including 1000 Genomes, ClinVar, and LOVD), fulfilling the PM2 criterion per ACMG/AMP guidelines. The frameshift variant is located in exon 3 of 5 exons, and NMDEscPredictor (https://nmdprediction.shinyapps.io/nmdescpredictor) predicts that the variant will lead to nonsense-mediated mRNA decay (NMD). Additionally, other pathogenicity prediction tools indicate that the frameshift variant disrupts the coding sequence, resulting in loss of protein function or dysfunction. Therefore, it is classified as a pathogenic variant (ACMG-PVS1) (Figure 3).

Functional studies indicate that RSPO4 loss-of-function variants impair Wnt/β-catenin signaling, disrupting dorsoventral limb polarity and nail morphogenesis (19). Murine models with conditional Rspo2 [R-spondin 2, which, like RSPO4 (R-spondin 4), belongs to the R-spondin protein family] knockouts recapitulate human anonychia (32, 33), further supporting pathogenicity. According to ACMG criteria (PVS1 + PM2-supporting), this variant was classified as “likely pathogenic” (clinically equivalent to pathogenic), confirming its diagnostic relevance.

## Discussion

Ectodermal Dysplasia (ED) is a genetic disorder characterized by the underdevelopment of ectodermal tissues, including teeth, hair, nails, and sweat glands (34). The primary underlying cause is single-gene variants (29). To date, over 200 distinct types of gene variants have been identified, originating from different chromosomes or genes encoding various proteins on the same chromosome (Jc et al., 2015). The clinical manifestations of ED are highly diverse, and individuals with different gene variants often exhibit varying phenotypes. Even different types of variants within the same gene can lead to significantly different clinical presentations. Anonychia, a form of ectodermal dysplasia, was first proposed by Cockayne in 1933 as a potentially recurrent inherited disorder (35). The secreted Wnt signaling ligand R-spondin 4 (RSPO4) is the first gene known to be associated with hereditary anonychia. Variants in this gene can lead to NDNC4, an autosomal recessive disorder. Researchers identified homozygous or compound heterozygous variants in the RSPO4 gene in 8 affected families and a German family (3, 4). Since this initial case, NDNC4 has been identified in diverse ethnicities worldwide (Table 1).

To explore the genetic and clinical landscape of NDNC4, our research included a comprehensive review of the literature concerning the clinical manifestations and genetic profiles of patients with RSPO4 variants (Table 1). Statistical analysis revealed that, to date, 22 families with NDNC4 have been reported worldwide. Through WES and Sanger sequencing, we identified a novel homozygous frameshift variant (c.268dupA/p.Cys91fs6) in the *RSPO4* gene. To the best of our knowledge, this is the first reported case of NDNC4 caused by the c.268dupA/p.Cys91fs6 frameshift variant in the *RSPO4* gene. It is also the first case in East Asia. This finding not only expands the genetic spectrum of RSPO4-related diseases but also enhances our understanding of the genetic basis of NDNC4.

**Figure 4:**
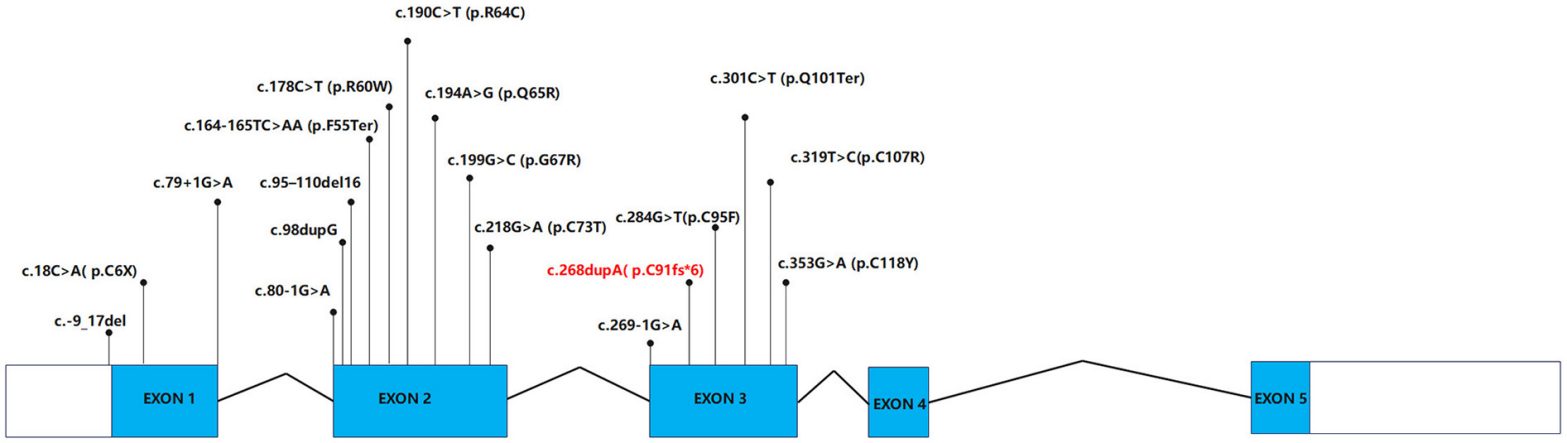
Pathogenic and likely pathogenic variants in the *RSPO4* gene. The schematic depicts the genomic structure of RSPO4 (exons 1–5) and variants in the *RSPO4* gene as recorded. Variants highlighted in red represent the novel frameshift variant (c.268dupA/p.Cys91fs*6) identified in this study.

Among patients with NDNC4, twenty-three families were found to carry 18 distinct variants in the *RSPO4* gene. The types of *RSPO4* variants involved included base deletions, nonsense variants, splice-site variants, and missense variants (see Table 1 and Figure 4). According to existing data in the literature, the nail phenotypes in individuals with NDNC4 are highly variable. The clinical manifestations range from reduced nail size to the complete absence of nail tissue, with some cases exhibiting missing or rudimentary nail primordia. Additional features include the absence of nail plates, swelling of the nail matrix, and protective hyperkeratosis of the nail bed. However, teeth and hair are typically normal, with no significant skin lesions observed. x-ray examinations of the hands and feet reveal the presence of terminal phalanges, and other systemic examinations show no abnormalities. The clinical presentation of the patients reported in this study is consistent with the characteristics of NDNC4. Nevertheless, genetic analysis remains the gold standard for diagnosing NDNC4. This case underscores the importance of clinical assessment combined with WES in diagnosing rare genetic disorders. Additionally, the case reported in this study exhibited non-specific phenotypic features, including short stature and hyperhidrosis. Further investigation is needed to determine whether these manifestations are directly linked to *RSPO4*.

As shown in Table 1, among 23 families carrying *RSPO4* variants, 20 exhibited homozygous variants, with 18 families involving consanguineous marriages—a practice that significantly increases the probability of homozygous recessive pathogenic alleles. Notably, the remaining two homozygous variant families had no documented consanguinity and were distributed across different regions. Given the low population frequency of this variant, the occurrence of homozygosity cannot be fully explained by random genetic transmission. Potential causes may include unidentified population migration backgrounds, regional founder effects (the region where these individuals are from may harbor additional affected individuals who have not been clinically ascertained), or environmental exposures. However, the most plausible explanation is undocumented distant consanguinity among the parents. To clarify the cause, it is necessary to determine whether there are multiple stretches of the genome that are identical by descent in both parents. However, unfortunately, only the patient underwent WES in this study, so we are unable to fully obtain the genetic information of the parents. In follow-up, we will conduct further investigations into their genetic background to better understand the potential causes and possible impacts on affected individuals.

Additionally, among the reported cases, both loss-of-function (LOF) and non-LOF variants have been found to cause NDNC4, with non-LOF variants accounting for 8 out of 18 cases. Notably, in the previously reported case F19, only a heterozygous *RSPO4* variant was identified, which contradicts the current understanding of the inheritance pattern. The authors of the article speculated that this could be due to mosaicism in the father or an undetected variant elsewhere in the *RSPO4* gene. Further exploration is needed to clarify these possibilities (36).

Different regions (domains) of R-spondin-4 serve distinct functions; two regions known as furin-like domains activate and stabilize proteins that play an indispensable role in the Wnt pathway.

To date, the majority of detected *RSPO4* variants have been concentrated in the highly conserved exons 2 and 3 (77.8%, 14/18), which affect the furin-rich cysteine domains in R-spondin 4, thereby impairing protein function. Some variants disrupt the structure of the furin-like domains, while others result in abnormally short proteins lacking these domains. Consequently, R-spondin-4 cannot participate in the Wnt signaling pathway, leading to abnormal or absent nail development. These findings suggest that the furin-like repeats encoded by exons 2 and 3 are considered essential for β-catenin activation and stabilization (13, 15, 37).

Further genotype-phenotype correlation studies have revealed that variants occurring in other domains may allow residual protein function, potentially resulting in a milder phenotypic expression, such as hyponychia or selective absence of single nails (2).

There is currently no medical or surgical treatment available for NDNC4, and artificial nails may be an option to improve appearance. However, due to financial reasons, the parents of the patient are not considering this option at present.Future therapeutic strategies would require further investigation into *RSPO4*-related molecular pathways.

## Conclusions

In this study, we identified a novel, likely pathogenic variant in the *RSPO4* gene of a Chinese girl with NDNC4 using WES, Sanger sequencing, and bioinformatics analysis. This finding broadens the known spectrum of *RSPO4* variants. Additionally, we systematically reviewed and analyzed the clinical and molecular genetic features of globally reported NDNC4 cases, thereby advancing our understanding of the genetic landscape of NDNC4 across diverse populations. This work provides insights for improving genetic diagnosis and clinical management of NDNC4, contributing to a deeper understanding of this rare inherited disorder.

We acknowledge the limitations inherent to a single-case study and emphasize the necessity for further research. Future studies should prioritize expanded sample sizes and integrate functional assays, such as *in vitro* and *in vivo* experiments, to elucidate the biological impact of the identified variant. Such studies will help elucidate the molecular mechanisms of NDNC4 and refine the genotype-phenotype correlations associated with *RSPO4* variants. Ultimately, these findings may facilitate more accurate genetic diagnosis, improved clinical management, and the development of targeted therapies for NDNC4, to optimize patient care and outcomes.

## Data Availability

The original contributions presented in the study are included in the article/Supplementary Material, further inquiries can be directed to the corresponding author.
